# Physiological Impacts on the Mosquito Vector Hosts Refine Vectorial Capacity Estimates of Mayaro Virus Transmission Risk

**DOI:** 10.3390/v17091155

**Published:** 2025-08-23

**Authors:** Luis A. Alonso-Palomares, John F. Williams, Edwin R. Burgess, John A. Lednicky, Rhoel R. Dinglasan

**Affiliations:** 1 Department of Infectious Diseases and Immunology, College of Veterinary Medicine, University of Florida, Gainesville, FL 32608, USA; luisalonsopaloma@ufl.edu (L.A.A.-P.); jfrederick.willi@ufl.edu (J.F.W.); 2 Emerging Pathogens Institute, University of Florida, Gainesville, FL 32610, USA; jlednicky@phhp.ufl.edu; 3 Entomology and Nematology Department, Institute of Food and Agricultural Sciences, University of Florida, Gainesville, FL 32608, USA; edwinburgess@ufl.edu; 4 Department of Environmental and Global Health, College of Public Health and Health Professionals, University of Florida, Gainesville, FL 32608, USA

**Keywords:** mayaro virus, *Anopheles gambiae*, *Anopheles albimanus*, *Aedes aegypti*, vector competence, survivorship

## Abstract

Mayaro virus (MAYV) is an alphavirus transmitted by mosquito vectors. Among the three MAYV genotypes (D, L, and N), genotype D has the broadest geographical distribution in Latin America and the Caribbean. The virus can be transmitted by the *Aedes*, *Anopheles*, and *Haemagogus* mosquitoes. To explore the potential expansion of MAYV across the Atlantic Ocean, we compared MAYV (D) infection kinetics in Floridian *Aedes* aegypti with New World (*Anopheles albimanus*) and Old World (*Anopheles gambiae*) anophelines. MAYV infection of both *An. albimanus* and *An. gambiae* was rapid, resulting in a higher dissemination rate than *Ae. aegypti*. We detected MAYV in saliva from *An. albimanus* (16.6% transmission rate) as early as 2 days post-infection (dpi), increasing to 60% after 7 dpi, a phenomenon (2 dpi) that has not been described to date for mosquitoes. We observed similar increases in MAYV infection of the ovaries and noted marked differences in fecundity for each species tested. Although MAYV infection in *An. gambiae* was rapid, mosquito lifespan was significantly reduced as compared with both *Ae. aegypti* and *An. albimanus*. We discuss the implications of our observations on MAYV transmission risk in Africa by *An. gambiae* and in the Caribbean and Central America by *An. albimanus*.

## 1. Introduction

Mayaro virus (MAYV; family *Togaviridae*, genus *Alphavirus*) is a mosquito-borne, single-stranded, positive-sense RNA virus that is the cause of Mayaro fever (MF). It was first isolated in 1954 from rural workers from Mayaro county in Trinidad and Tobago [1]. Phylogenetic studies indicate that there are at least three MAYV genotypes: D (widely dispersed), L (limited), and N (new). Genotype D has the greatest geographical distribution, as it has been reported in Argentina, Brazil, Colombia, Peru, Suriname, Trinidad and Tobago, and Venezuela [2]. The symptoms of MF are like those of other arboviral infections caused by chikungunya virus (CHIKV), dengue virus (DENV), and Zika virus (ZIKV) [3,4]. Since all these arboviruses can circulate concurrently in the same area, misdiagnosis rates are high [5,6]. In total, there have been more than 900 cases of MAYV infection reported in Latin America, and this number continues to increase [7]. MAYV has a sylvatic transmission cycle between *Haemagogus* spp. mosquitoes and non-human primates, birds, rodents, sloths, and other small animals [8]. Due to this sylvatic cycle, MAYV infections appear to be highest among human populations that work in forested areas [9]. The virus has also been associated with other mosquito vectors including species of the *Aedes*, *Anopheles*, and *Culex* genera [10]. Since urban-dwelling *Aedes* and *Culex* mosquitoes are also competent vectors, the public health risk of a transition from sylvatic to urban transmission cycles increases [11,12], which may result in the subsequent spread of arboviruses beyond endemic regions through travel and commerce. Among the New World anophelines, *An. albimanus* is responsible for the transmission of malaria in Central America, the northern region of South America, and the Caribbean. Its distribution overlaps with endemic MAYV transmission, and it was recently reported to be competent for MAYV under laboratory conditions [13]. Among the Old World anophelines, *An. gambiae,* the primary vector responsible for malaria transmission in Sub-Saharan Africa, is the most well-studied. Both *An. albimanus* and *An. gambiae* have been shown to be competent vectors for alphaviruses such as the O’nyong-yong virus and MAYV [14,15]. The broad geographic range of several competent New- and Old-World mosquito vector genera increases the potential risk of the introduction of MAYV beyond the Americas [11,13,16]. Although laboratory studies have previously demonstrated vector competence for MAYV, to date, a closer analysis of the impact of virus infection on vector physiology and how this can impact MAYV transmission risk by different vector species remains limited [13]. Here, to develop a better understanding of MAYV transmission risk by mosquitoes, we used the MAYV Uruma strain (D genotype) to infect *Ae. aegypti*, *An. albimanus*, and *An. gambiae* strains to further characterize the kinetics of virus dissemination from a blood meal and its impact on vector host physiology.

## 2. Methods

### 2.1. Cell Lines

Cell lines used for virus propagation were obtained from the American Type Culture Collection (ATCC, Manassas, Virginia, USA). Vero E6 [*Cercopithecus aethiops* (African green monkey) kidney cells, ATCC CRL 1586] and BHK-21 cells [*Mesocricetus auratus* (Syrian golden hamster) kidney cells, ATCC CCL-10] were propagated as monolayers at 37 °C in 5% CO_2_ in complete Dulbecco’s Modified Medium, high glucose (DMEM, Gibco Cat. 11965118), containing 10% fetal bovine serum (FBS, Gibco Cat.16140071), 1 × penicillin/streptomycin (Gibco Cat. 15140122), and a final concentration of 2 mM/L-Glutamine (Gibco Cat. A2916801) until 80% confluence prior to virus infections.

### 2.2. Mosquito Maintenance

The *Ae. aegypti* (Orlando, ORL) and *An. albimanus* (STECLA) eggs were provided by the United States Department of Agriculture (USDA), Agricultural Research Service (ARS) Center for Medical, Agricultural and Veterinary Entomology (CMAVE). The *An. gambiae* (KEELE) mosquitoes were a generous gift from P. Eggleston and H. Hurd from Keele University, United Kingdom. Mosquito eggs were hatched in deionized water (DI), and larvae were grown at a density of 200 larvae/tray. Adult mosquitoes were maintained on 10% sucrose solution while housed in 28 °C growth chambers at 80% relative humidity (RH) and a 12/12 light/dark cycle at the University of Florida as per standard procedures [17]. To propagate the colonies, females were fed (1:1) O+ human red blood cells (RBCs) and human heat-inactivated serum (HHIS) in a water-jacketed artificial membrane feeder set to 38 °C.

### 2.3. Virus Culture and Plaque Assay

Vero E6 cells at 80% confluence were infected with the MAYV Uruma strain (Bolivia, 1959), genotype D (BEI Resources), using fresh DMEM with 3% FBS. The culture flasks were monitored every day until virus-induced cytopathic effects (CPE) were detected. The cell culture medium was collected and centrifuged at 600× *g* at 4 °C for 5 min to pellet debris. The virions in the supernatant were then stabilized by the addition of trehalose solution to a final concentration of 10% (*w*/*v*) trehalose (Sigma Aldrich, St. Louis, MO, USA, Cat. T0167) before storage in liquid nitrogen. The titer of the virus stocks was obtained by plaque assay using BHK-21 cells. Briefly, the cells were seeded onto the wells of a 24-well plate, and after the cells had adhered and reached confluence, serial 10-fold dilutions of the virus preparation were performed. The cells were inoculated with 200 μL of reduced serum medium (3% FBS plus L-glutamine) containing a 1:10 dilution of the virus and then incubated for 1 h at 37 °C in a 5% CO_2_ atmosphere. Subsequently, 1 mL of 0.8% methylcellulose (Sigma Aldrich, Cat. M0512) was resuspended in DMEM medium, and 1X penicillin/streptomycin was added to each plate without removing the previously added virus. The plates were then incubated for 3 days at 37 °C in a 5% CO_2_ atmosphere. After 3 days, the semi-solid medium was removed. The wells were then stained using 1 mL of a 1:1 mixture of methanol and acetone with 1% crystal violet for 30 min. Excess dye was gently washed off with tap water, and the virus plaques were counted to calculate plaque forming units/mL (PFU/mL).

### 2.4. Mosquito Infection and Tissue Collection

The mosquitoes were fed a blood meal containing a mixture of 2:2:1 O+ human RBCs, 1 × 10^7^ PFU/mL of MAYV, and HHIS in an Arthropod Containment Level 3 facility. Engorged, the infected mosquitoes were maintained on 10% sucrose at 28 °C, 80% RH up until the time of sample collection. Prior to dissections, the mosquitoes were cold anesthetized at 20 °C for 10 min and kept on ice until processing. To extract saliva, the legs and wings were removed, and the proboscis was placed into a capillary tube for 30 min, containing 10 µL of 1 volume of human RBC and 3 volumes of HHIS [18]. The midgut, ovaries, abdominal carcasses (minus midgut and ovaries), and head–thorax were collected at 2, 7, and 14 days post-infection (dpi). All samples were placed in 1.5 mL safe-lock microfuge tubes (Eppendorf Cat. 0030123328) containing 500 µL of DMEM medium with 2% FBS, 1× penicillin/streptomycin, and 0.15 g of 0.5 mm glass beads (Next Advance Cat. GB05). Samples were stored at −80 °C until processed.

To determine the infection rate (IR), we counted the number of MAYV-positive midgut samples over the total number of mosquitoes that were provided with an infectious blood meal. To estimate the dissemination rate (DR), we counted the number of virus-positive head–thorax, abdominal carcass (minus midgut), and paired ovary samples over the number of infected midguts. The transmission rate (TR) is based on the total number of virus-positive saliva samples over the total number of virus-infected mosquitoes. The transmission efficiency (TE) is the proportion of all female mosquitoes that had MAYV-positive saliva over the total number of infectious blood-fed females.

### 2.5. RNA Extraction and RT-qPCR

The samples were thawed on ice and homogenized in a Bullet Blender^®^ (Cat. BT24M) at speed 8 for 5 min. The samples were then centrifuged at 3800× *g* for 3 min at 4 °C to pellet debris. RNA was extracted from the virions in the supernatant using a QIAmp Viral RNA Mini Kit (Qiagen, Cat. 52906), following the manufacturer’s instructions. Specific oligonucleotides were used to amplify MAYV nsP1, and the conserved housekeeping gene ribosomal protein L32 (RNA extraction control). Primer information can be found in Supplementary Table S1. The quantitative reverse transcription polymerase chain reaction (RT-qPCR) was performed using the UltraPlex 1-Step ToughMix (4X) from QuantaBio (Avantor Cat. 95166-500). Each sample was processed in duplicate using the Azure Cielo Real-Time PCR system (Azure Biosystems Mod. AIQ060). The thermocycling program consisted of an initial step at 50 °C for 10 min, followed by 95 °C for 2 min, and then 45 cycles of 95 °C for 15 s and 60 °C for 45 s. Samples were considered positive if the quantification cycle (Cq) was <38. Amplification controls were included on each plate from two biological replicate studies using duplicate replicates per sample.

### 2.6. Estimating Vector Survivorship

Mosquitoes were provided with a MAYV-infected blood meal (1 × 10^7^ PFU/mL) in a 30 cm × 30 cm × 30 cm (single-fed) cage before being transferred into pint (473 mL) ice cream cups (N = 25 mosquitoes/cup) and separated into two subgroups for the survivorship study. These cups were monitored daily to record survival rates until 14 dpi (dead mosquitoes were removed). At 4 dpi, one of the two infected groups was provided with uninfected human blood (double-fed) to determine if a second blood meal would influence survivorship. A control group of mosquitoes that were fed only with blood, either once or twice, was included in the study. After 14 dpi, the amount of viral genome in the remaining live mosquitoes was analyzed in the infected groups to determine the prevalence of infection. Virus infection prevalence was also performed for dead mosquitoes removed from the cups during daily monitoring. Survivorship assays were conducted in duplicate.

### 2.7. Fecundity Measurements

Approximately 250–300 females (5–7 days old) were collected into four 473 mL cups. The females were starved for about 6 h before being blood fed via an artificial membrane feeder according to their assigned experimental group. After blood feeding for 30 min, the mosquitoes were knocked down at 4 °C for 10 min. Two groups of 100 engorged females were transferred to a 20 cm × 20 cm × 20 cm cage (two cages total). Two days after the blood meal, an oviposition cup containing a 90 mm Whatman filter cone (egg paper) was placed inside the cages containing the *An. albimanus* and *An. gambiae* mosquitoes. For *Ae. aegypti*, the oviposition cup was placed three days after the introduction of the infected blood meal. An oviposition cup containing a 90 mm Whatman filter cylinder was placed. The *Anopheles* oviposition cups were removed the next day, and the *Aedes aegypti* oviposition cups were removed after two days. The egg paper was placed flat on a paper towel in a tray to dry. An image of the egg paper was acquired (2532 × 1170 pixel resolution) using a 12 MP iPhone camera, 20 cm above the paper, and with default camera settings. The eggs were counted using the ImageJ 1.53t (August 24, 2022; https://imagej.net/ij/index.html) software.

### 2.8. Statistical Analyses

For vector competence assays, we used one-way ANOVA with Tukey’s post hoc test on pairwise comparisons to compare tissue or mosquito infection prevalence and two-way ANOVA for comparing the number of blood meals and viremia within and between species for each of the two biological replicate studies performed. These tests were performed using the GraphPad Prism 10.4.1 software, considering significance at a *p*-value of <0.05. Statistical significance annotation used a common letter (e.g., ‘a’ and ‘a’) to indicate not statistically significant differences (α  =  0.05), whereas different letters (e.g., ‘a’ and ‘b’) indicated statistically significant differences. For survival analyses, Cox regression on MAYV-fed mosquitoes was performed using R version 4.3.2 [19] and the ‘survival’ package [20]. Data was visually inspected using Kaplan–Meier curves, and the assumption of proportional hazards across the measurement window was assessed using Schoenfield residuals with the ‘coxzph’ function. For the analysis of egg counts between the control and treatment groups, we used an unpaired t-test with Welch’s correction (i.e., unequal variances assumed). Statistical significance of pairwise comparisons among the treatments and control was determined at a *p*-value of <0.05.

## 3. Results

### 3.1. Rapid Dissemination of MAYV in Mosquitoes Is Genus-Specific

**Figure 1:**
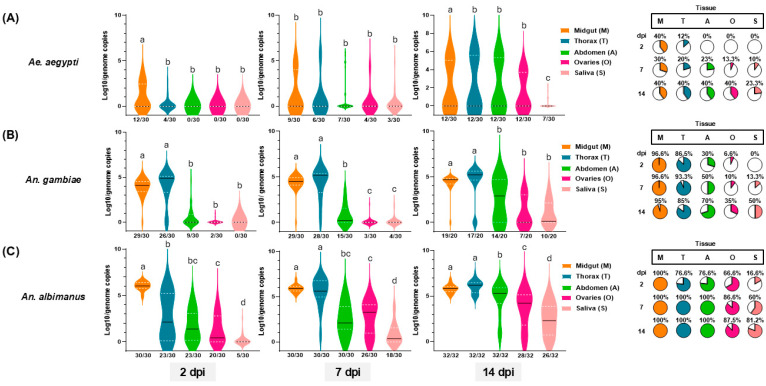
Vector competence of *Ae. aegypti* (**A**), *An. gambiae* (**B**), and *An. albimanus* (**C**) for MAYV. Mosquitoes were orally infected at the same multiplicity of infection (MOI). Midgut, thorax (including head), abdomen (minus midgut and ovaries), ovaries, and saliva samples were collected at 2, 7, and 14 days post-infection (dpi). RNA was extracted from each of these tissues to measure the prevalence of infection for each species by RT-qPCR. Samples with Cq ≤ 38 were considered positive. The *x*-axis for each time point and tissue indicate the number of positive samples/total number of samples. The black horizontal line indicates the median genome copies, and the dashed lines indicate the quartiles. Tissue-specific genome copies annotated with a common letter (e.g., two datasets sharing a common ‘a’ or common ‘b’) were found to be not statistically significant (*α*  =  0.05). The proportion of infected tissues are shown on the pie charts to the right. Summary data are provided in Table 1.

To measure the vector competence of *Ae. aegypti*, *An. gambiae,* and *An. albimanus* mosquitoes for MAYV-D, we orally infected mosquitoes for each species (N = ~30/species) and dissected tissues at 2, 7, and 14 dpi. While the 7 and 14 dpi time points are historically evaluated when determining vector competence for an arbovirus, we included the 2 dpi time point, as it has been shown that arbovirus infection kinetics and virus–vector interactions can occur much earlier [17]. At 2 dpi, we observed rapid infection and partial dissemination in *Ae. aegypti* wherein the IR was 40% and DR was 33.3% but limited to only the head–thorax samples, as no infections were observed in the abdominal carcass, ovaries, or saliva (Figure 1A, Table 1). At 7 dpi, the midgut IR was 30% and DR was 66.6% in the head–thorax, 77.7% in the abdomen, and 44.4% in the ovaries. We also observed a TR of 33.3%, with detection of MAYV in saliva. The estimated overall TE was 10% (Figure 1A, Table 1). By 14 dpi, the midgut IR remained constant at 40%; however, the DR increased to 100% for the head–thorax, abdominal carcass, and ovaries. As expected, the TR increased to 58.4% and the IR increased up to 23.3% (Figure 1A, Table 1).

For *An. gambiae,* we observed a higher midgut IR (96.6%) and a broader tissue DR including the head–thorax (89.6%), abdominal carcass (31%), and ovaries (6.8%) at 2 dpi (Figure 1B, Table 1). We did not observe MAYV in saliva samples at this time point. The midgut IR remained constant at 96.6% at 7 dpi, and the DR increased to 96.5% in the head–thorax, 51.7% in the abdominal carcass, and 10.3% in the ovaries. The observed TR (13.7%) was comparable to *Ae. aegypti* at 14 dpi; however, the TE was 13.3% (Figure 1B, Table 1). At 14 dpi, the midgut IR at 7 dpi was 90%, but the DR decreased for the head–thorax (89.4%) and abdominal carcass (73.6%), while the DR increased for the ovaries (36.8%). We also observed a TR of 52.6% (Figure 1B, Table 1), which differed from previous infection studies with MAYV, genotype D, and the *An. gambiae* KEELE strain [21].

We also observed that *An. albimanus* is highly susceptible to MAYV infection with high IR, DR, and TR estimates at 2 dpi. We observed a midgut IR of 100% and a DR of 76.6% for the head–thorax and abdominal carcass, and 66.6% for the ovaries. However, in contrast to *An. gambiae*, we noted a TR of 16.6% at this early time point (Figure 1C, Table 1). At 7 and 14 dpi, the DR increased, reaching 100% for the head–thorax and abdominal carcass (Figure 1C, Table 1). For the ovaries, the DR was comparable, going from 86.6% at 7 dpi to 87.5% at 14 dpi. The TR rose from 60% at 7 dpi to 81.2% at 14 dpi (Figure 1C, Table 1).

Finally, we compared the TE for each vector and observed that for *Ae. aegypti*, the TE at 2 dpi was 0% and increased to 10% at 7 dpi, reaching 23.3% at 14 dpi. For *An. gambiae* infections, we observed results similar to those for *Ae. aegypti*, with a TE of 0% and 13.3% at 2 and 7 dpi, respectively. However, by 14 dpi, the TE increased to 50%. Interestingly, for *An. albimanus*, the transmission efficiency (TE) reached 16.6% at 2 dpi. This increased to 60% at 7 dpi, similar to *An. gambiae* at the same time point, and rose to 81.2% at 14 dpi (Table 1).

### 3.2. Differential Vector Survivorship Following MAYV Infection

**Figure 2:**
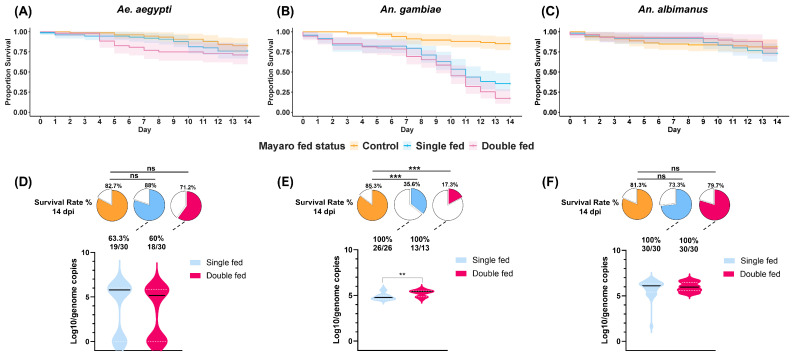
Survivorship of *Ae. aegypti*, *An. gambiae,* and *An. albimanus* following MAYV infection. Three cohorts of each mosquito species were analyzed. The evaluated conditions were (i) mosquitoes that were exclusively fed a non-infectious blood meal (represented by below), (ii) mosquitoes fed an infectious blood meal (blue line), and (iii) mosquitoes that were first fed with an infectious blood meal and subsequently provided a second non-infectious blood meal at 4 dpi (purple line). Survivorship was monitored until 14 dpi (**A**–**C**). The figures below illustrate the survival rates after 14 dpi for the three experimental groups, as well as the number of mosquitoes testing positive for MAYV infection: *Ae. aegypti* (**D**), *An. gambiae* (**E**), and *An. albimanus* (**F**). Two replicate studies were performed. Annotations: ns, not statistically significant, ** indicates *p* < 0.01 and *** indicates *p* < 0.001.

While conducting vector competence studies, we observed a consistent drop in the survival of MAYV-infected *An. gambiae* at 14 dpi, which had not been previously described in the literature. To investigate whether MAYV infection differentially impacts the lifespan of *Ae. aegypti*, *An. gambiae*, and *An. albimanus*, we repeated the mosquito infections using the same MOI with the inclusion of a second non-infectious blood meal and performed survival analysis. Across the measurement interval, 14 dpi, the hazard rate of mortality (i.e., hazard ratios [HR]) of infected *Ae. aegypti*, whether single-fed or double-fed, were not significantly different from the controls or between single-fed or double-fed conditions (Figure 2A; Supplementary Figure S1). We observed that survival of *Ae. aegypti* infected with MAYV was reduced by 22% after 14 dpi in single-fed mosquitoes and by 28.8% in double-fed compared with the controls. For infected *An. gambiae*, single-fed mosquitoes had a statistically significant 6.6-fold increased hazard rate of mortality relative to the control. For double-fed *An. gambiae*, there was a greater, and statistically significant, 9.7-fold increase in the hazard rate of mortality relative to the control. The double-fed *An. gambiae* also had a statistically significant 1.5-fold increased hazard rate of mortality relative to the single-fed mosquitoes (Figure 2B). At 14 dpi, survivorship was reduced by 64.4% in single-fed mosquitoes, and 82.7% in double-fed mosquitoes. Similarly to *Ae. aegypti*, and in contrast to *An. gambiae,* we found no differences in the hazard rate of mortality regardless of fed status or control with *An. albimanus* survival (Figure 2C).

We then measured MAYV infection prevalence to determine if it was associated with survivorship outcomes for each mosquito species cohort. For *Ae. aegypti*, we observed that live mosquitoes that were fed once with a MAYV-infected blood meal had an infection prevalence of 63.3% while mosquitoes that were provided with a second, but uninfected blood meal had a comparable prevalence of 60% (*p* = 0.1065) (Figure 2D). For *An. gambiae* and *An. albimanus* infections, we observed 100% infection among the mosquitoes that were alive and fed once or twice at 14 dpi (Figure 2E,F). To determine if mosquito death was associated with a higher infection prevalence and viremia (i.e., virus genome copies), we examined the infection prevalence among the deceased mosquitoes that were fed once vs. twice at 14 dpi. Dead *Ae. aegypti* that fed once had an infection prevalence of 66%, while those that fed twice showed a significantly lower prevalence of 21.4% (*p* < 0.001), although viremia among infected samples in each group was not statistically significant (*p* = 0.0663) (Supplementary Figure S2A). For *An. Gambiae*, we observed 100% infection prevalences for both live and dead mosquitoes. However, live mosquitoes that were blood fed twice had a statistically significant difference in viremia (*p* <0.05) (Figure 2E), whereas for dead *An. Gambiae*, the viremias were lower in double-fed mosquitoes (*p* < 0.05) **(**Supplementary Figure S2B). For *An. Albimanus*, we observed 100% infection prevalence in surviving and dead mosquitoes and no statistically significant difference in viremia among live (*p* = 0.8426) or dead (*p* = 0.7468) mosquitoes fed either once or twice (Figure 2F; Supplementary Figure S2C).

### 3.3. Vector Species-Specific Fecundity Impacts Following MAYV Infection

**Figure 3:**
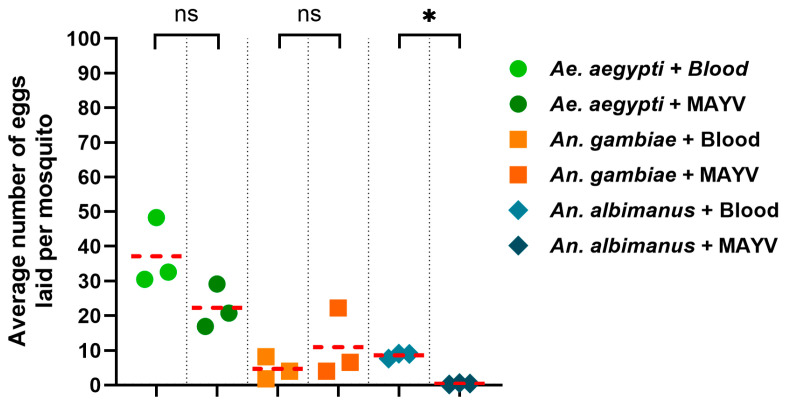
Ovary infection with MAYV impacts *Ae. aegypti*, *An. Gambiae*, and *An. albimanus* fecundity. Mosquitoes were orally infected and provided with an oviposition cup at 2 dpi to collect eggs. The number of eggs laid per group of female mosquitoes is shown. Three replicate studies (N = 100 mosquitoes/group/replicate) were performed. The thick horizontal line indicates the mean egg number. Annotations: ns, not statistically significant and * indicates *p* < 0.05.

To determine if MAYV impacted fecundity, we repeated the same infections and collected egg clutches. A control group that only received blood was included in each experimental group for each species. We observed that MAYV decreased the number of eggs laid by *Ae. aegypti* mosquitoes as compared with the control group, but this difference was not statistically significant (*p* = 0.1028). *An. gambiae* laid a comparable number of eggs (*p* = 0.3901) between the infected and the controls, while the infected *An. albimanus* suffered a significant reduction in the number of eggs laid as compared with the uninfected group (*p* < 0.05) (Figure 3). These data indicate that MAYV dissemination to ovaries following initial infection results in a significant species-specific difference in fecundity.

## 4. Discussion

We have shown that MAYV can be transmitted by saliva within a short period of incubation by anophelines, which are infrequently incriminated vectors for arboviruses, especially MAYV. It is widely recognized that MAYV is transmitted in urban areas by mosquitoes of the *Aedes* genus. However, there has been little research focused on other species, particularly *Anopheles* spp. with overlapping geographic distribution with *Ae. aegypti*. To date, we know little about the physiological impact of the virus on the vector, and how these impacts can influence MAYV transmission risk. Despite an observed IR of 40% for *Ae. aegypti* (ORL strain) as early as 2 dpi, the virus was not detected in saliva. In contrast, previous MAYV vector competence studies with *Ae. aegypti* mosquitoes (MAYV/BR/Sinop/H307/201) reported an IR of 61.3% in salivary glands at 3 dpi [12]. However, that study used a MAYV-L genotype and did not directly detect MAYV in the secreted saliva [12]. In another study using *Ae. aegypti* collected in Miami, FL, and infected with MAYV-D (TRVL 4675; Trinidad & Tobago, 1954), the prevalence of infection in the saliva at 3 dpi was less than 5%, which increased to approximately 8% at 6 dpi and increased further up to 30% at 12 dpi [8]. Our results showed an increase in the TR up to 33.3% at 7 dpi, and up to 58.3% at 14 dpi. The same study reported an IR of up to 60% at 3 dpi using the full-body approach, with only a slight increase observed at 12 dpi. In contrast, our study showed an IR of 40% at 2 dpi, which remained unchanged by 14 dpi (Figure 1A, Table S1). These data suggest that the lab-reared *Ae. aegypti* (ORL) are more competent than wild-caught *Ae. aegypti* from Miami, FL, for MAYV-D. In a separate study, *Ae. aegypti* mosquitoes collected from Vero Beach, FL, which were orally infected with the same MAYV-D (TRVL 4675) at a temperature of 30 °C, had an IR (whole bodies) of ~30% at 3 dpi, which increased to 60% at 15 dpi [22]. They detected MAYV-D in saliva in < 5% of the mosquitoes at 3 dpi, but the infection prevalence in the saliva increased to 16% at 15 dpi [22]. These findings are comparable to our results. Although the vector competence experiments used *Ae. aegypti*, we know that there are vector host genetic variations that mediate vector competence for arboviruses [23]. It is possible that both field and lab strains of *Ae. aegypti* that are derived from Florida are intrinsically less competent for MAYV, as previously hypothesized [24,25].

Although previous studies have explored the MAYV infection of both *An. gambiae* and *An. albimanus* [13,14], the DR of the virus at earlier time points and in various tissues in the vector was not fully determined. We observed that although *An. gambiae* has a high IR of 96.6%, we did not detect MAYV in its saliva, making transmission unlikely at this early time point (Figure 1B, Table 1). In contrast, for *An. albimanus*, we observed an IR of up to 100% at 2 dpi, and surprisingly, the mosquitoes were capable of transmitting MAYV in saliva at this time point, with a TE of 16.6% (Figure 1C, Table 1). Previous infections of *An. gambiae* (G3 strain) with MAYV-D (BeAn343102, Brazil, 1978) reported an IR of 75% at 7 dpi, with no virus was detected in the saliva [14]. In another previous study using the *An. albimanus* STECLA strain, MAYV IR was 100% and the TE was up to 20% at 7 dpi, which is the earliest time in that study [13]. Notably, at 7 dpi, we observed an IR of 96.6% and a TE of 13.3% for *An. gambiae* and an IR of 100% and a significantly higher TE of 60% for *An. albimanus*. For these other studies, the IRs for both *An. gambiae* and *An. albimanus* increased up to 100% [13,14]. However, the TE for *An. albimanus* at 14 dpi only increased by 2-fold to 48% [13], as compared with a TE of 100% for *An. gambiae*, albeit for a small number of analyzed mosquitoes (N = 4) [14]. In our study, the IR remained consistent for both mosquito species at 14 dpi. However, the TE increased significantly, reaching 50% for *An. gambiae* (a 3.7-fold increase over 7 dpi), and 81.2% for *An. albimanus* (a 1.4-fold increase over 7 dpi). It should be noted that while the *An. albimanus* STECLA strain was common to the previous study and the study herein, the *An. gambiae* strains were different. The G3 and KEELE strains are known to be quite different in their competence for malaria parasites, and G3 was recently determined to be an interspecific hybrid of *An. coluzzii* and *An. gambiae* [26].

MAYV genotype is likely a major factor influencing IR, DR, and TE in different mosquito vectors. For example, the high viremias reported in other studies for *Ae. aegypti* at 7 dpi or 14 dpi were associated with the L genotype [24,25], albeit the TE was low or non-existent. We demonstrated that *An. gambiae* (KEELE) and *An. albimanus* (STECLA) are permissive to infection with the MAYV genotype D (Uruma strain), achieving TEs of 50% for *An. gambiae* and 81.2% for *An. albimanus* at 14 dpi, indicating a significant risk of transmission. We hypothesize that intra-genotype variation within MAYV-D may account for the contrasting results of the study herein using the Uruma strain (D genotype) from previous studies using the BeAn343102 strain (D genotype).

Vectorial capacity (VC) measures how effectively an arthropod vector can transmit a pathogen from an infected individual to a new, susceptible host in both disease-endemic and non-endemic foci. The key factors considered in the VC equation are human-biting rate, vector longevity, the extrinsic incubation period (EIP) of the pathogen (the time to develop before it can be transmitted to a new host), and vector density (reproductive fitness). We measured survivorship to assess how MAYV infection affects the lifespan of multiple vectors. In our study, we observed that MAYV-infected *Ae. aegypti* mosquitoes did not exhibit any significant differences in lifespan compared with the control group (Figure 2A). Additionally, there is evidence suggesting that a second ingestion of a non-infectious blood meal can enhance virus escape from the midgut [27], but it was unclear if increased viremias would result in life shortening [28]. Since both *Aedes* and *Anopheles* can take multiple blood meals during their EIP and natural gonotrophic cycle, we included a group that received a second non-infectious blood meal. We did not observe any significant differences between the double-fed group and single-fed conditions for *Ae. aegypti* (Figure 2A). However, for *An. gambiae*, we observed significant differences in survivorship between the single-fed group and both the double-fed and control groups (Figure 2B). In contrast, when comparing between groups of *An. albimanus*, we found no significant differences in survivorship (Figure 2C). Previous survival analyses showed that infection with both MAYV-D (BeAn343102) and MAYV-L (BeAr505411) significantly affected the lifespan of *An. albimanus* [13]. These results are opposite to our findings; however, the genotype D used in that study was different from ours (Uruma strain). This may occur due to genetic variations between different viral genotypes and the genetic background of the mosquito, leading to variations in vector fitness and competency. In summary, these data suggest that MAYV-D infections affect survivorship in a vector species-specific manner.

We also assessed the prevalence and viremia in live MAYV-infected mosquitoes in our survivorship analyses at 14 dpi. We did not observe any differences in infection prevalence or viremia between the single-fed and double-fed groups, which differs from a report using the same *Ae. aegypti* (ORL) strain infected with MAYV-L, indicating that a second blood meal increases the prevalence of infection in the midgut and subsequent tissue dissemination. This enhancement is more pronounced in the early days of infection. However, the differences diminish over time, and by 10 dpi, the disparity becomes less significant [29]. These results at the later time point align with our findings at 14 dpi wherein we did not observe any differences in infection prevalence or viral genome load. For *An. gambiae,* we observed no differences in infection prevalence but noted a statistically significant difference in viremia between single-fed and double-fed mosquitoes, a viremia difference that was not observed for *An. albimanus*. Among the mosquitoes that died during the survival analysis study, only the *Ae. aegypti* groups showed any statistically significant difference in infection prevalence and viremia between the single-fed and double-fed groups. Considering that there was a lower infection prevalence and viremia among double-fed mosquitoes, MAYV infection was not a driving factor for low survivorship for *Ae. aegypti*. Therefore, in addition to survivorship, we also report on vector species-specific differences in infection and viremia associated with survival.

We initially observed MAYV dissemination to the ovaries for *Ae. aegypti* only at 7 dpi, reaching a DR of 100% at 14 dpi (Table 1). In contrast, for *An. gambiae*, we observed a DR of 6.8% at 2 dpi, which increased to 36.8% at 14 dpi. Interestingly, *An. albimanus* had a high ovarian DR of 66.6% at 2 dpi, peaking at 87.5% at 14 dpi. Overall, MAYV infection of *Ae. aegypti* (ORL) resulted in significantly fewer eggs laid, half the number of controls (Figure 3), consistent with earlier reports of Florida mosquitoes infected with MAYV genotype D [29], albeit a different strain, whereas *An. gambiae* fecundity was not affected by MAYV infection; *An. albimanus* showed virtually no egg production (Figure 3). It is likely that the high ovarian DR of >40% within 7 dpi results in arrested egg maturation and reduced oviposition.

Taken together, these data provide additional insights into the vectorial capacity of these three vectors species for MAYV, which has direct implications on transmission dynamics and outbreak risk. The prototypical success of *Ae. aegypti* for the urban transmission of MAYV is due in part to a slower virus DR and TE and no significant impacts on survivorship or fecundity. Rapid MAYV dissemination to ovaries and saliva within a week in *An. albimanus* does not affect survival but results in a significant reduction in fecundity, this indicates a direct suppression of the population, which is not sustainable. We can therefore reasonably speculate that a MAYV outbreak driven by *An. albimanus* may be initially intense, but short-lived due to population suppression. These factors may explain in part how *An. albimanus* has remained an underappreciated vector for MAYV for so long [13]. As infected *An. albimanus* do not produce offspring, resulting in a reduction in adult population size, the mosquitoes are therefore unsampled by entomological surveys that are conducted well after detection of the human index case. On the other hand, the introduction of MAYV to Sub-Saharan Africa and subsequent transmission by *An. gambiae* may result in an intense but also prolonged outbreak. Despite the reduction in survivorship at 7 dpi, the shorter EPI for MAYV in *An. gambiae* results in larger numbers of mosquitoes becoming infectious at a younger age. Moreover, as it is a highly anthropophilic vector that takes frequent blood meals in a single gonotrophic cycle [30], the resulting increase in viremia and dissemination may lead to an even larger infectious adult female population that is not suffering from reduced fecundity. While these findings raise important public health concerns about the introduction of MAYV to the African continent, they also reveal opportunities for using MAYV as a tool to explore life-shortening and fecundity-reducing mechanisms that can inform the design of novel biological control approaches.

## Figures and Tables

**Table 1:** Summary of infection rate (IR), dissemination rate for each tissue (DR), and transmission rate (TR) and transmission efficiency (TE) for each species of mosquito infected with MAYV (Uruma, Genotype D) at 2, 7, and 14 dpi depicted in Figure 1.

	Days Post-Infection (dpi)	IR	DR Head–Thorax	DR Abdomen	DR Ovaries	TR	TE
***Ae. aegypti***	2 dpi	40.0% 12/30	33.3% 4/12	0.0% 0/12	0.0% 0/12	0.0% 0/12	0.0% 0/30
	7 dpi	30.0% 9/30	66.6% 6/9	77.7% 7/9	44.4% 4/9	33.3% 3/9	10.0% 3/30
	14 dpi	40.0% 12/30	100.0% 12/12	100.0% 12/12	100.0% 12/12	58.3% 7/12	23.3% 7/30
***An. gambiae***	2 dpi	96.6% 29/30	89.6% 26/29	31.0% 9/29	6.8% 2/29	0.0% 0/29	0.0% 0/30
	7 dpi	96.6% 29/30	96.5% 28/29	51.7% 15/29	10.3% 3/29	13.7% 4/29	13.3% 4/30
	14 dpi	90.0% 19/20	89.4% 17/19	73.6% 14/19	36.8% 7/19	52.6% 10/19	50.0% 10/20
***An. albimanus***	2 dpi	100.0% 30/30	76.6% 23/30	76.6% 23/30	66.6% 20/30	16.6% 5/30	16.6% 5/30
	7 dpi	100.0% 30/30	100.0% 30/30	100.0% 30/30	86.6% 26/30	60.0% 18/30	60.0% 18/30
	14 dpi	100.0% 32/32	100.0% 32/32	100.0% 32/32	87.5% 28/32	81.2% 26/32	81.2% 26/32

## Data Availability

All datasets and reagents generated through this study are available from the corresponding author upon reasonable request through a material transfer agreement.

## Supplementary Materials

The following supporting information can be downloaded at https://www.mdpi.com/article/10.3390/v17091155/s1, Table S1: Oligonucleotide primer sequences used for RT-qPCR synthesis. Figure S1: Survival tables for *Ae. aegypti*, *An. gambiae*, and *An. stephensi.* Single-fed mosquitoes refer to mosquitoes that received only a single Mayaro virus (MAYV)-infected blood meal. Double-fed mosquitoes refer to those that received a single MAYV-infected blood meal and then a subsequent non-infectious blood meal. Refer to the main text for more details. Figure S2: MAYV infection prevalence among dead mosquitoes that were fed once with an infectious blood meal only, and mosquitoes fed with an infectious blood meal and then followed up with a non-infectious blood meal. (**A**) *Ae. aegypti*. (**B**) *An. gambiae*. (**C**) *An. albimanus*. Horizontal bar indicates the median genomic copy number, and dashed lines indicate the quartiles. Asterisks depict statistical significance at alpha = 0.05. Representative data are shown.
