# Supplementary material for: Physiological Impacts on the Mosquito Vector Hosts Refine Vectorial Capacity Estimates of Mayaro Virus Transmission Risk
Source: Viruses. 2025 Aug 23;17(9):1155. doi: 10.3390/v17091155 (PMC12474012; doi:10.3390/v17091155)
Supplement: Supplementary file 1 [file viruses-17-01155-s001.zip › viruses-3746466-supplementary.pdf]

# SUPPLEMENTAL DATA

### TITLE: Physiological impacts on the mosquito vector hosts refine vectorial capacity estimates of Mayaro virus transmission risk

## SUPPLEMENTARY TABLES

**Supplementary Table S1.** Oligonucleotide primer sequences used for RT-qPCR synthesis.

<table>
<tr><th>Gene</th><th>Accession No.</th><th>Sequence (5' - 3')</th><th>Size (bp)</th></tr>
<tr><td rowspan="3">MAYV (nsp1)</td><td rowspan="3">MN138459.1</td><td>5’-CCTTCACACAGATCAGAC-3’</td><td rowspan="3">96</td></tr>
<tr><td>5’-GCCTGGAAGTACAAAGAA-3’</td></tr>
<tr><td>5’-CATAGACATCCTGATAGACTGCCACC-3’</td></tr>
<tr><td rowspan="3">Ae. aegypti L32</td><td rowspan="3">LOC5577996<br>(XM_001656684)</td><td>5’-CAGTCCGATCGCTATGACAA-3’</td><td rowspan="3">200</td></tr>
<tr><td>5’-ATCATCAGCACCTCCAGCTC-3’</td></tr>
<tr><td>5’-ACGTTGTGGACCAGGAACTT-3’</td></tr>
<tr><td rowspan="3">An. gambiae L32</td><td rowspan="3">LOC1281371<br>(XM_320915)</td><td>5’-GCCGAAGATTGTGAAGAAGC-3’</td><td rowspan="3">220</td></tr>
<tr><td>5’-GACGTTGTGGACCAGGAACT-3’</td></tr>
<tr><td>5’-GCGCACCCGATTGTCAATACCTTT-3’</td></tr>
<tr><td rowspan="3">An. albimanus L32</td><td rowspan="3">LOC118457443<br>(XM_035919035)</td><td>5’-GAGTAAGAGGAGAAGGAGGAGTAG-3’</td><td rowspan="3">136</td></tr>
<tr><td>5’-CTTTGGTTTGTGCGGATCATC-3’</td></tr>
<tr><td>5’-ATCAGCGATTGGTGTTGTGCTGC-3’</td></tr>
</table>

<u>**SUPPLEMENTARY FIGURES**</u>

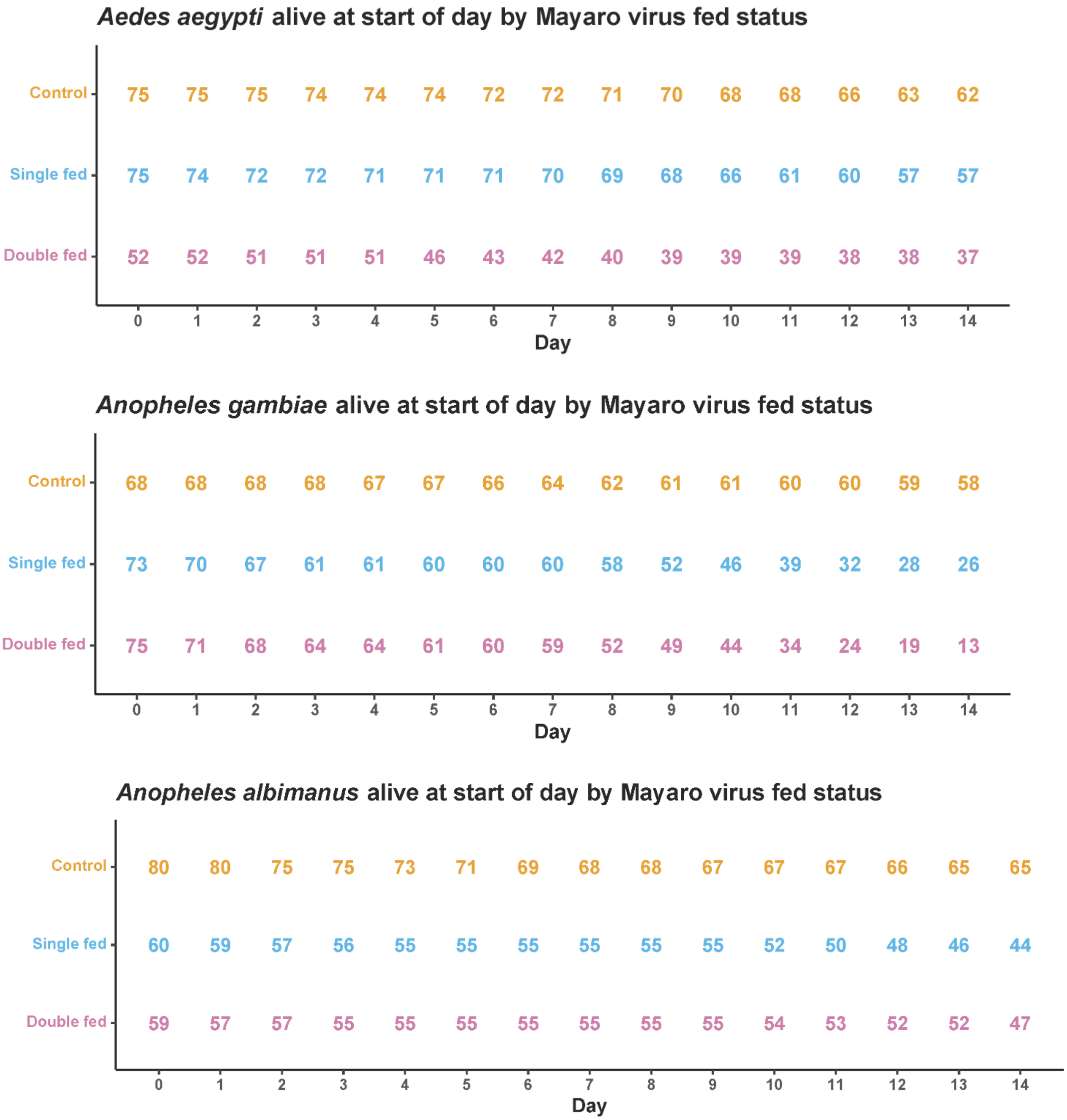


**Supplementary Figure S1. Survival tables for *Ae. aegypti, An. gambiae,* and *An. albimanus.*** Single-fed mosquitoes refer to mosquitoes that received only a single Mayaro virus (MAYV)-infected blood meal. Double-fed mosquitoes refer to those that received a single MAYV-infected blood meal and then a subsequent non-infectious blood meal**.** Refer to the main text for more details.

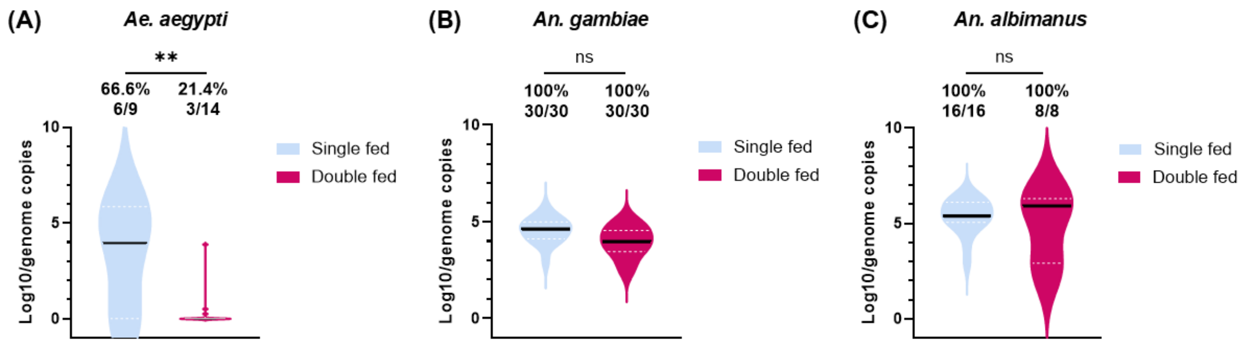


**Supplementary Figure S2. MAYV infection prevalence among dead mosquitoes that were fed once with an infectious bloodmeal only, and mosquitoes fed with an infectious bloodmeal and then followed up with a non-infectious bloodmeal.** (A) *Ae. aegypti*. (B) *An. gambiae*. (C) *An. albimanus*. Horizontal bar indicates the median genomic copy number, and dashed lines indicate the quartiles. Asterisks depict statistical significance at α= 0.05. Representative data are shown.
